# Embolization with MVP (Micro Vascular Plug^®^): experience on 104 patients in emergent and elective scenarios

**DOI:** 10.1186/s42155-021-00246-2

**Published:** 2021-07-12

**Authors:** Francesco Giurazza, Anna Maria Ierardi, Andrea Contegiacomo, Fabio Corvino, Giampaolo Carrafiello, Raffaella Niola

**Affiliations:** 1 Interventional Radiology Department, Cardarelli Hospital, Via Cardarelli 9, 80100 Naples, Italy; 2 Radiology Department, Fondazione IRCCS Cà Granda Ospedale Maggiore Policlinico, Via Della Commenda 10, 20100 Milan, Italy; 3 Fondazione Policlinico Universitario A. Gemelli IRCCS, Largo Agostino Gemelli 8, 00136 Rome, Italy

**Keywords:** Micro Vascular Plug, Embolization, Hemorrhage, Arterial

## Abstract

**Aim:**

To describe a 3 years experience of peripheral arterial embolization with Micro Vascular Plug (MVP) (Medtronic, USA).

**Materials and methods:**

The following parameters were investigated: type of vascular injury, anticoagulation therapy at time of procedure, anatomical district, caliper of the target artery, course of the landing zone, additional embolics, technical and clinical success, device related clinical complications. Technical success was defined as complete embolization without deployment of additional embolics after MVP release. Primary clinical success was considered as hemodynamic stability in emergency setting and resolution of the underlying vascular pathology in elective cases; secondary clinical success was considered clinical success after a second embolization session.

**Results:**

116 MVP have been released in 104 patients (67 males and 37 females; mean age 61.3 years). The pullback release technique was adopted in each case. 85 patients were treated in emergent settings while in 19 patients the procedure was scheduled. The overall technical success was 75%. Primary clinical success was 96.1%, secondary clinical success 3% and clinical failure 0.9%. No statistical differences in terms of effectiveness were observed among patients assuming anticoagulation (*p*-value = 0.6). A straight and longer landing zone were statistically associated with higher technical success compared to curved and shorter ones, (*p*-values < 0.001 and = 0.048 respectively). MVP-3 and MVP-5 were the most frequently adopted models in this sample, in 29.8% and 49% of the patients respectively. No clinically adverse events directly related to MVP occurred; in 3 cases device migration was registered without clinical complications.

**Conclusion:**

MVP is a safe and effective embolic agent. While eventual concomitant anticoagulation therapy did not influence the technical outcome, straight course and length of the landing zone are essential parameters to evaluate before deployment.

## Background

The Micro Vascular Plug (MVP) (Medtronic, USA) is a mechanical embolic device with control detachment, made of a self-expanding nitinol skeleton ovoid-shaped covered with a polytetrafluoroethylene (PTFE) coating and soldered to a pusher wire (Giurazza et al. 2018). The models MVP-3 and MVP-5 are delivered through a microcatheter (0.027″), MVP-7 and MVP-9 requires a diagnostic catheter (4Fr and 5Fr respectively).

The first experience description in literature is relatively recent, dated 2014 (Pellerin et al. 2014); since then, few papers (Abdelsalam et al. 2020; Bailey et al. 2019; Barrett et al. 2018; Boatta et al. 2017; Conrad et al. 2015; Duvnjak et al. 2018; Giurazza et al. 2019; Jardinet et al. 2020; Mahdjoub et al. 2018; Ratnani et al. 2019), have reported on the use of MVP in interventional radiology focusing on small samples of patients, especially affected by pulmonary arteriovenous malformations.

This study aims now to describe a 3 years experience of arterial embolization with MVP in both emergent and elective scenarios on a large sample; patients characteristics and landing zone features have been analyzed to identify parameters that may influence the technical outcome.

## Materials and methods

This is a multicenter retrospective observational study; the local ethical committees approved the study. All patients treated in elective conditions gave their written informed consent to the procedure; those managed in emergency signed a written consent in case their clinical conditions allowed.

Local electronic records have been analyzed to detect all patients that underwent to a transarterial embolization using MVP between 1 January 2018 and 31 December 2020.

Both emergent and elective procedures have been considered.

The following parameters have been investigated: age, sex, underlying pathology, type of vascular injury, anticoagulation therapy at time of procedure, anatomical district, caliper of the target artery, course of the landing zone, additional embolics, technical and clinical successes.

In all cases a multiphasic contrast-enhanced CT was acquired before the procedure. In emergent setting, the procedural indication was based on the hemodynamic status and on the CT findings (active bleeding, hematoma, pseudoaneurysms, arteriovenous-fistula). In elective scenario, the procedural indication derived from a multidisciplinary evaluation of the underlying disease and on the lesion characteristics evaluated at CT (size, district, inflow-outflow etc.).

Technical success was considered as complete vessel occlusion at final digital subtraction angiography (DSA) without subsequent deployment of additional embolics.

Primary clinical success was evaluated after embolization accomplishment with MVP and other eventual embolic agents; it was intended as hemodynamic stability with increased/stabilized hemoglobin values in emergent scenarios and as resolution of the underlying vascular pathology in elective patients; secondary clinical success was considered clinical success after a second embolization session.

Data analysis according to each MVP model size has been conducted also.

Clinically adverse events directly related to MVP device were classified according to CIRSE classification system for complications (Filippiadis et al. 2017).

### Release technique

**Fig. 1:**
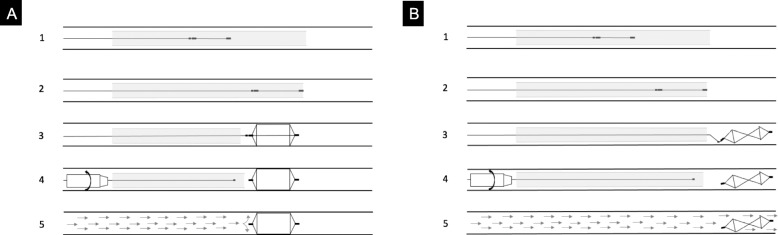
Schematic drawing of MVP release technique. In **A**, the correct pull-back technique is reported; in step 1 MVP is positioned into the catheter (proximal and distal radiopaque markers are indicated by long black segments while the radiopaque detachment point by short black segment); in step 2 MVP is advanced up to the tip of the catheter; in step 3 the catheter is moved backward in order that MVP is completely expanded outside the catheter; in step 4 MVP is detached with mechanical torqueing; in step 5 MVP properly occludes the vessel lumen with the blood flaw being interrupted. In **B**, the wrong pushing technique is reported; in steps 1 and 2, as in A, MVP is positioned into the catheter and advanced up to the tip of the catheter; in step 3 MVP is pushed forward outside from the catheter, with its nitinol skeleton kinked and crushed not properly expanded; in step 4 MVP is detached with mechanical torqueing; in step 5 MVP is wrongly released not interrupting the blood flow into the vessel lumen

The MVP has been always released with the pull-back technique (Giurazza et al. 2018) avoiding the pushing technique (Fig. 1): the microplug is first advanced in order that the distal radiopaque marker is positioned in correspondence of the distal tip of the catheter; then, the catheter is withdrawn up to the detachment zone and finally the MVP is released. In case the operators judged necessary and feasible, a fluoroscopic check has been performed to verify MVP expansion before detachment.

All attempts have been applied to place the pusher wire in axis with the MVP in order that the detachment zone was in a straight position, not angled. MVP-3 and MVP-5 were released through a 2.7 Fr microcatheter while MVP-7 and MVP-9 through a 5 Fr diagnostic catheter.

Before introducing the MVP through the hub, the whole catheter/microcatheter dead space has been flushed with saline in order to avoid clots formation that could hinder the release.

The target vessel caliper was measured at preprocedural CT scan in arterial phase or at diagnostic DSA. Based on previous literature experience (Giurazza et al. 2019), the MVP models were empirically oversized 30% in elective and 40% in emergent patients.

### Statistical analysis

Chi-square test was employed to investigate the relationship between technical success and ongoing anticoagulant therapy, MVP landing zone course and MVP landing zone length respectively; *p*-value was considered significant if < 0.05.

ROC curves analysis was performed to assess possible cut-off values of vessel diameter which could predict technical success for MVP-3 and MVP-5, according to the area under the curve (AUC); this was unfeasible for MVP-7 and MVP-9 due to the low number of cases.

Electronic database was conducted with Excel^®^ Micosoft Corp. (USA); descriptive and inferential statistical analysis were performed using SPSS^®^ v.22 IBM (USA).

## Results

116 MVP have been released in 104 patients (Table 1): in 93 patients one MVP, in 10 patients two MVP and in one patient three MVP.

**Table 1:** Overall sample data

N. of Pts	N. of MVP	Mean age (range)	Sex	Anticoag. tx	LZ course	LZ length vs MVP
104	116	61.3 (20–90)	37F 67M	34 yes 70 no	30 curved 86 straight	15< 101>

N.: number; Pts: patients; Anticoag.: anticoagulation; tx: therapy; LZ: landing zone

The overall mean age of the sample was 61.3 years (range: 20–90) and it was composed of 67 males and 37 females.

30 MVP (25.9%) have been released in a curved arterial segment while 86 (74.1%) in a straight arterial segment; 15 MVP (12.9%) have been deployed in a vessel segment shorter than the MVP length while 101 MVP (87.1%) in longer one.

In 85 patients the embolization has been performed in emergency while in the other 19 patients the procedure was scheduled (Table 2).

**Table 2:** Underlying pathologies according to emergent and clinical scenarios

Clinical scenario	Pathology
Emergent	57 traumatic 19 spontaneous 5 bleeding neoplasms 4 bleeding duodenal ulcers
Elective	11 AVF 6 splenic aneurysms 2 pre-surgical (renal tumor resection)
Total	104

AVF: arterio-venous fistula

34 patients (32.7%) were under anticoagulation therapy at the time of the embolization: all of them presented in emergency setting with hemorrhages, 19 spontaneous and 15 post-traumatic.

Vessel occlusion was achieved in 78 patients after MVP release, while in 26 subjects additional embolics were required; therefore the overall technical success was 75%. Primary clinical success was 96.1%, secondary clinical success 3% and clinical failure 0.9%.

Concerning anticoagulation therapy at the time of the procedure, no statistical differences (*p*-value = 0.6) were observed in terms of technical success between patients assuming and not assuming: among the 78 patients with technical success, 24 were under anticoagulation therapy at the time of the procedure; among the 26 patients with technical failure, 10 assumed anticoagulation therapy.

A straight landing zone was statistically associated with higher technical success compared to a curved one (*p*-value < 0.001); among the 78 patients with technical success, the landing zone was straight in 67 and curved in 11; among the 26 patients with technical failure, the landing zone was straight in 7 and curved in 19.

Furthermore, a landing zone longer than the unsheated MVP length was associated with higher technical success compared to a shorter one (*p*-value = 0.048); among the 78 patients with technical success, the landing zone was longer in 71 and shorter in 7; among the 26 patients with technical failure, the landing zone was longer in 18 and shorter in 8.

Overall, MVP-3 and MVP-5 were the most frequently adopted model in this sample, in 29.8% and 49% of the patients respectively.

### MVP-3 group analysis

**Fig. 2:**
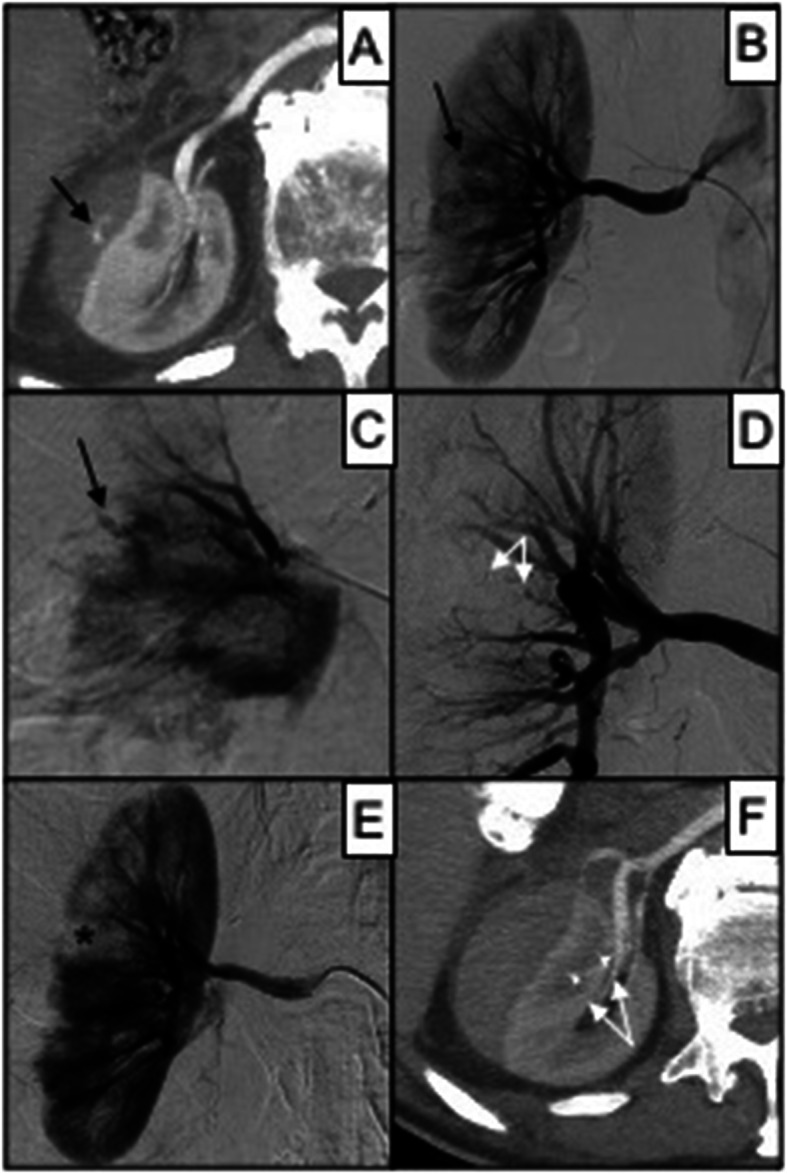
86 years old male affected by renal bleeding after car investment. In **A**, a contrast-enhanced CT scan in arterial phase shows perirenal hematoma of the right kidney refurnished by active bleeding (black arrow); in **B**, DSA confirms active bleeding (black arrow) from a distal intraparenchymal branch of the right renal artery; in **C**, superselective DSA shows the bleeding vessel (black arrow); in **D**, superselective DSA shows bleeding resolution after MVP-3 release (target vessel caliper 1.3 mm); in **E**, renal DSA confirms proper embolization with minimal ischemic area (black asterisk); in **F**, day after CT scan follow-up shows effective embolization without MVP (white arrows) metallic artifacts

34 MVP-3 have been adopted in 31 patients (Table 3). The target arteries were: segmental hepatic (2), bronchial (2), intercostal-lumbar (2), inferior epigastric (3), division branch of the renal (4) (Fig. 2), hypogastric branches (4), gastrointestinal (4) and limbs vessels (13).

**Table 3:** Results according to MVP model considering number of devices deployed, mean target vessel caliper, technical success and primary clinical success

MVP model	N. of patients treated	Mean target vessel caliper in mm (range)	Technical success (%)	Primary clinical success (%)
MVP-3	31	2.1 (0.6–2.8)	67.6	90.3
MVP-5	51	3.2 (1.1–5)	61.4	98
MVP-7	11	4.5 (2.7–5.1)	91.7	100
MVP-9	11	6.5 (2.7–8)	69.2	100

N.: number; mm: millimeter

The target vessel caliper was in mean 2.1 mm (range: 0.6–2.8).

13/34 MVP (38.2%) were released after other embolic failed to obtain vessel occlusion.

Technical success was obtained in 23/34 patients (67.6%); in 11 cases, additional embolics were required (coils in 6 patients, spongel slurry in 4 cases and glue in 1 case).

Primary clinical success was obtained in 28 patients (90.3%), secondary clinical success in 2 (6.5%); one patient had clinical failure with death for hypovolemic shock (3.2%).

**Fig. 3:**
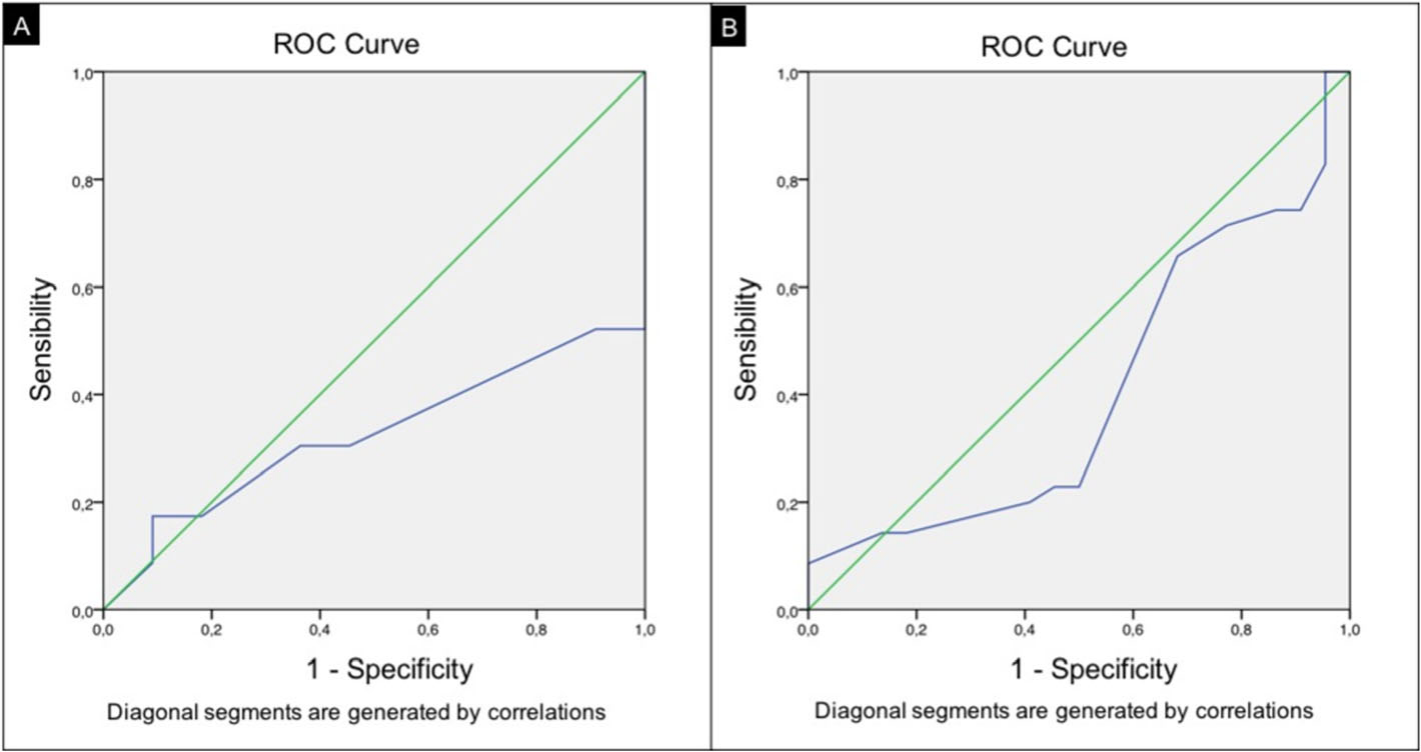
ROC curve analysis for MVP-3 (**A**) and MVP-5 (**B**)

The ROC curve analysis showed a slight trend to technical success in case the MVP was deployed in a vessel with a caliper < 2.1 mm (AUC: 0.326) (Fig. 3a).

### MVP-5 group analysis

**Fig. 4:**
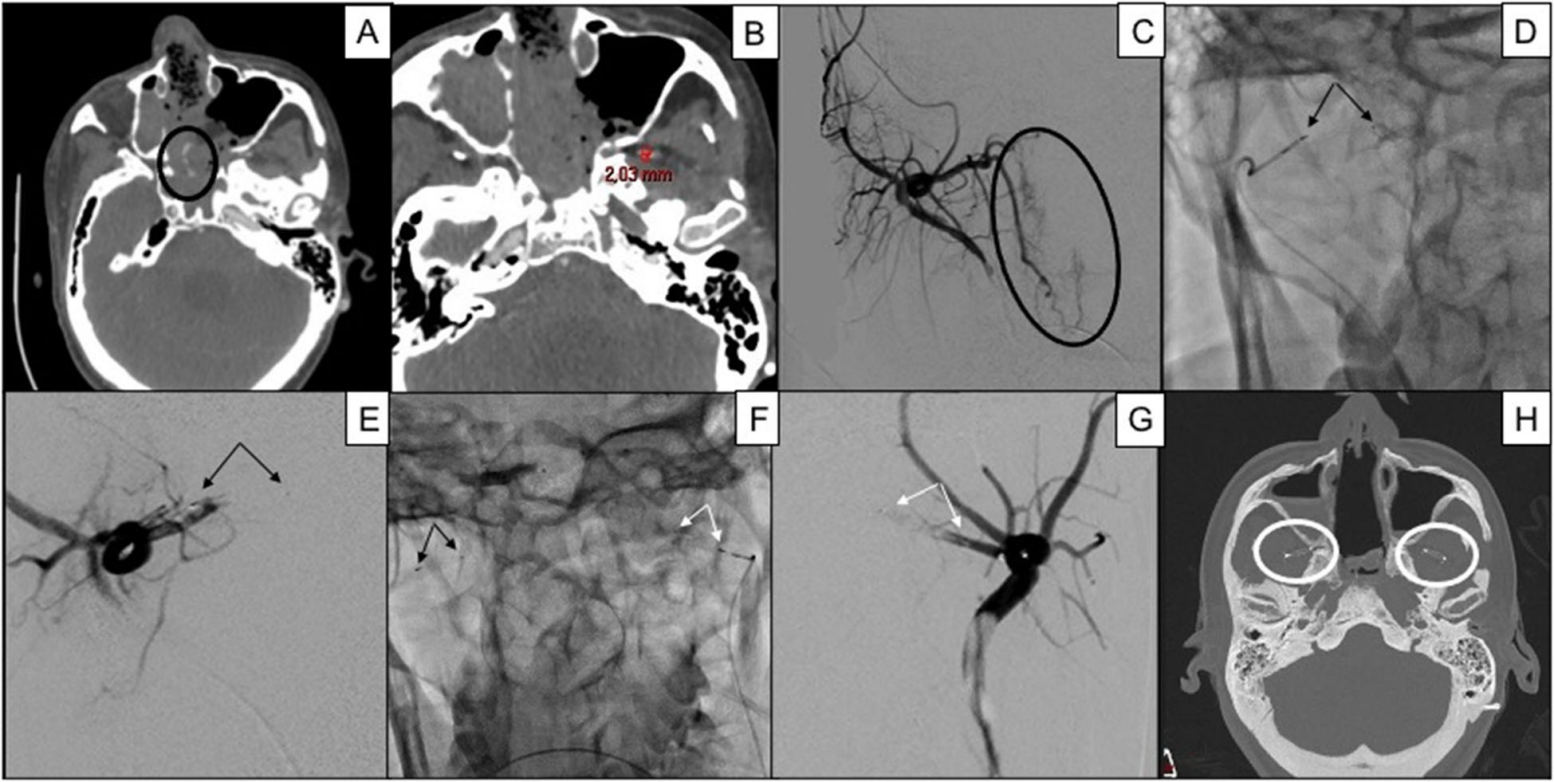
37 years old male affected by bleeding nasopharyngeal carcinoma after radiotherapy; bilateral internal maxillary artery embolization was performed, first with 300–500 microparticles and then, because of continuous bleeding, two MVP-5. In **A**, a contrast-enhanced CT scan in arterial phase shows active bleeding (black circle); in **B**, the target vessel internal maxillary artery is measured with caliper 2.03 mm, embolization being performed bilaterally; in **C**, right internal maxillary DSA confirms active bleeding (black circle); in **D**, MVP-5 is released through a 2.7 Fr microcatheter into the distal segment of the right internal maxillary artery, black arrows indicating distal and proximal radiopaque markers: in **E**, DSA confirms proper right internal maxillary artery occlusion immediately after MVP release; in **F**, a second MVP-5 (white arrows) is similarly positioned into the left internal maxillary artery (black arrows indicating MVP-5 previously released); in **G**, DSA confirms proper left internal maxillary artery occlusion immediately after MVP release; in **H**, an axial CT scan of the skull shows the two MVP-5 (white circle) not creating any metallic artifact

57 MVP-5 have been adopted in 51 patients (Table 3). The target arteries were: splenic (1), pulmonary branches (2), gastrointestinal (2), bronchial (2), segmental hepatic (3), external carotid branches (4) (Fig. 4), intercostal-lumbar (7), inferior epigastric (8), hypogastric branches (8), division branch of the renal (9) and limbs vessels (11).

The target vessel caliper was in mean 3.2 mm (range: 1.1–5).

31/57 MVP (54.4%) were released after other embolic failed to obtain vessel occlusion.

Technical success was obtained in 35/57 patients (61.4%); in 22 cases, additional embolics were required (coils in 12 patients, spongel slurry in 7 cases and glue in 3 cases).

Primary clinical success was obtained in 50 patients (98%), secondary clinical success in the other one (2%).

The ROC curve analysis showed a slight trend to technical success in case the MVP was deployed in a vessel with a caliper < 3.1 mm (AUC: 0.405) (Fig. 3b).

### MVP-7 group analysis

**Fig. 5:**
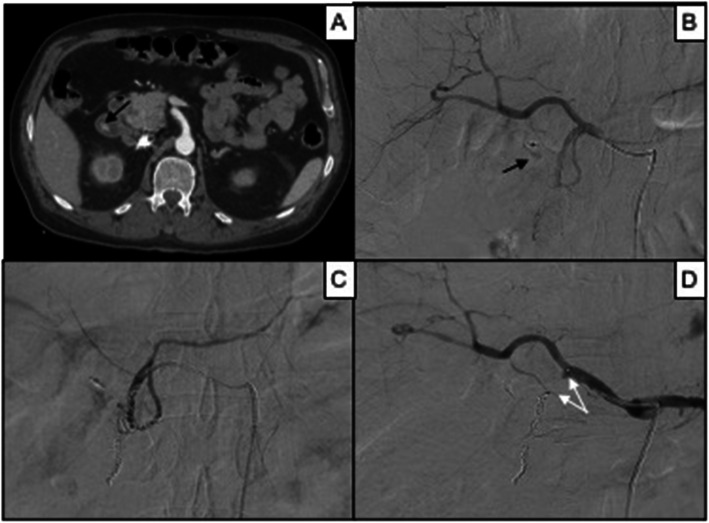
67 years old male affected by bleeding duodenal ulcer; previous attempts to manage the patient endoscopically failed. In **A**, a contrast-enhanced CT scan in arterial phase shows active intraluminal bleeding (black arrow); in **B**, DSA confirms active bleeding (black arrow) from the gastroduodenal artery; in **C**, DSA shows uneffective coiling of the gastroduodenal artery with blood flow continuing into the vessel; in **D**, DSA shows complete gastroduodenal artery occlusion after release of MVP-7 (white arrows) in correspondence of the origin of the vessel (target vessel caliper 4.1 mm)

12 MVP-7 have been adopted in 11 patients (Table 3). The target arteries were: splenic (1), limbs vessel (1), gastroduodenal (2) (Fig. 5), inferior epigastric artery (3) and renal trunk (5).

The target vessel caliper was in mean 4.5 mm (range: 2.7–5.1).

6/12 MVP (50%) were released after other embolic failed to obtain vessel occlusion.

Technical success was obtained in 11/12 patients (91.7%); in 1 case additional embolics were required (coils).

Primary clinical success was obtained in all patients (100%).

### MVP-9 group analysis

13 MVP-9 have been adopted in 11 patients (Table 3). The target arteries were: hepatic (1), external carotid (1), gastroduodenal (1), limbs vessel (2), pulmonary branches (2) and splenic (6).

The target vessel caliper was in mean 6.5 mm (range: 2.7–8).

1/13 MVP (7.7%) was released after other embolics failed to obtain vessel occlusion.

Technical success was obtained in 9/13 patients (69.2%); in 4 cases additional embolics were required (coils in 3 patients and glue in one patients).

Primary clinical success was obtained in all patients (100%).

### Patients treated in emergency setting

Among the 85 patients treated in emergency, 57 had post-traumatic hemorrhages, 19 had spontaneous bleedings assuming anticoagulation therapy, 5 had bleeding neoplasms and 4 had bleeding duodenal ulcers.

Technical success was obtained in 54 (63.5%); additional embolics after MVP release were required in the other 31 patients (coils in 15, spongel slurry in 11, glue in 4, another MVP in one).

Primary clinical success rate was achieved in 81 subjects (95.3%). In four patients bleeding recurred, requiring re-embolization: of these, three patients had secondary clinical success while one patient died.

Concerning the model size adopted, MVP-3 was selected in 31 subjects, MVP-5 in 43, MVP-7 in 8 and MVP-9 in 3.

**Fig. 6:**
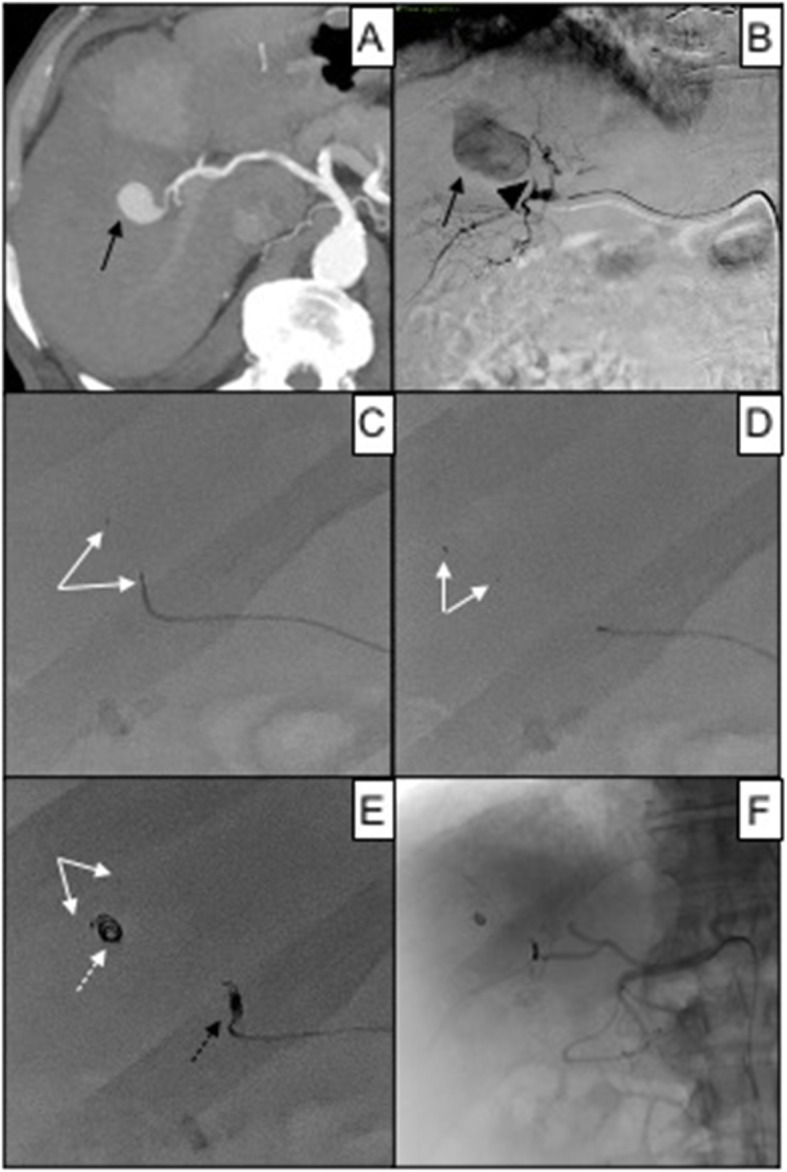
83 years old female affected by iatrogenic pseudoaneurysm of the right hepatic artery after RFA for HCC. In **A**, a contrast-enhanced CT scan in arterial phase shows the intraparenchymal vascular lesion (black arrow); in **B**, superselective DSA of the right hepatic artery confirms pseudoaneurysm (black arrow) refurnished by a short arterial feeder (black arrowhead); in **C**, attempt to perform MVP-3 (white arrows) embolization, target vessel caliper 1.1 mm; in **D**, due the shortness of the arterial feeder, MVP-3 (white arrows) migrated into the pseudoaneurysm sac; in **E**, proper embolization was obtained with a 2 mm Concerto^®^ Medtronic controlled detachment coil (black dotted arrow), after that another pushable coils (white arrow) migrated into the pseudoaneurysm sac together with MVP (white arrow); in **F**, fluoroscopic control showing resolution of the pseudoaneurysm

According to CIRSE classification system for complications (Filippiadis et al. 2017), no clinically adverse events directly related to MVP device occurred. Three MVP migrated distally after release; in one patient the landing zone was shorter than the unsheated MVP (Fig. 6), while in the other two the landing zone was curved in splenic artery.

## Discussion

In this study MVP allowed to obtain a complete vessel occlusion in 75% of the cases. While anticoagulation regimen uptake at the time of the intervention did not influence the device embolic property (*p*-value = 0.6), statistical significant correlations with technical success have been found concerning the landing zone characteristics: a straight vessel (*p*-value < 0.001) longer than the unsheated MVP (*p*-value = 0.048).

The main technological improvement provided by MVP is the possibility to release a plug peripherally; models MVP-3 and MVP-5, which were the most adopted model in this sample (29.8% and 49% of the patients respectively), present the relevant advantage of microcatheter compatibility. Apart from their size, this is possible thanks to the high flexibility of nitinol skeleton and pusher wire, which allow navigability into diagnostic catheters and microcatheters without creating tension. Empirically, even in thin and tortuous vessels, MVP does no induce retreat and instability of the delivering catheter.

In order to obtain a correct release of the device, the pullback technique preceded by saline flush of the catheter dead space is mandatory. Another relevant aspect to consider is the landing zone evaluation; in this sample a straight landing zone was significantly associated with technical success. This is related to the nitinol skeleton proper expansion, producing effective adhesion of the PTFE covering to the vessel wall; furthermore, the torqueing detachment is facilitated because the detachment point is on the same long axis of the MVP. Also, the length of the landing zone proof to be significantly related to technical success; this should be longer than the MVP length to allow the expansion of the full device without risk of distal migration, as in the case described in Fig. 6.

In this study the choice of MVP model was empirically oversized 30% in elective and 40% in emergent patients according to previous literature experience (Giurazza et al. 2019); especially in bleeding patients, vasospasm may underestimate the vessel diameter measurement. Oversize allows the proper expansion of the nitinol skeleton, ensuring adherence of the PTFE covering to the vessel wall. For MVP-3 and MVP-5 a trend to technical failure has been analyzed for target vessel calipers higher than 2.1 mm and 3.1 mm respectively.

Concerning patients assuming anticoagulation therapy, in this study the embolic property of MVP was not influenced by this factor; compared to coils, this advantage should be related to the PTFE covering which create a full lumen physical barrier to the blood flow when it properly adheres to the arterial wall. Indeed the technical failure rate (25%) observed in this study was related mainly to a curved and/or short landing zone and not to anticoagulation status. Another advantage of this device compared to other metallic embolics is the absence of artifacts at follow-up CT.

Published series have already demonstrated the effective occlusive property of MVP in both cranial (Kleine et al. 2015; Carlson et al. 2017; Burkhardt et al. 2018; Shwe et al. 2014; See et al. 2017) and extracranial vascular embolization procedures (Boatta et al. 2017; Conrad et al. 2015; Ratnani et al. 2019; Mahdjoub et al. 2018; Duvnjak et al. 2018; Bailey et al. 2019; Barrett et al. 2018; Giurazza et al. 2018, 2019); its applications included both emergent and elective conditions. Among others, multiple experiences about renal hemorrhages embolization reported satisfying results (Giurazza et al. 2019; Jardinet et al. 2020); they reported complete embolization with a single MVP in 80% and 66% respectively, these values being similar to the technical success rate reported in our study. Concerning the elective scenarios, MVP seems to present particular advantages for occlusion of pulmonary arteriovenous malformations (Boatta et al. 2017; Bailey et al. 2019; Barrett et al. 2018; Conrad et al. 2015; Duvnjak et al. 2018; Mahdjoub et al. 2018; Ratnani et al. 2019), with technical success ranging between 91 and 100%. In our study we have applied MVP to occlude pulmonary arteriovenous fistula in 4 patients, with MVP-5 and MVP-9 in two cases each; in three patients MVP completely occluded the fistula while in one case additional coils were necessary. Some authors applied this device also to obtain a temporary flow diversion during a radioembolization procedure (Abdelsalam et al. 2020). Another paper described MVP application to occlude the neck of a common femoral artery pseudoaneurysm (Talaie et al. 2020). Its use has been even described in pediatric patients affected by congenital heart diseases, especially for the closure of patent ductus arteriosus (Boudjemline 2017; Wang-Giuffre and Breinholt 2017; Sathanandam et al. 2017). Similar to our experience, in all these studies no device related major complications have been described; however, we have encountered three MVP distal migration without clinical sequelae (one hepatic and two splenic arteries). Description of an extravascular field of application has been reported in a case report where a thoracic duct leak was occluded with MVP (Chick et al. 2017). However, all these experiences have included a small number of patients and did not perform an analysis to identify which parameters may influence the technical outcome.

Even if a comparison with coils was not part of the aim of this paper, MVP should not be considered as an alternative to them. Apart from a different higher mean cost, it presents specific features: its predictive landing zone makes it particularly interesting in case deployment is close to vascular bifurcation or to spare healthy vessels. In patients assuming anticoagulation, PTFE covering makes it an effective embolic. Finally, if the size is properly selected, a single MVP allows effective and immediate occlusion; usually to obtain embolization with coils, more than one are released and time to obtain clot formation needs to be waited.

This paper presents some limitations. First, its design is retrospective observational: having analyzed a lapse of 3 years, the operators have improved their skills in that time and so technical failure would be more frequent in the first part of the study period. Moreover, heterogeneity in clinical practice among the two centers may represent a confounding factor in interpreting the results. Then, the sample is disomogeneous because included emergent and elective conditions; this may induce bias in the proposed results because of the different hemodynamic status: vasoconstriction and flow-dynamic influence the choose of the MVP model size; however, this study aims to report an overall experience of embolization with this device rather than focusing on a specific scenario.

## Conclusions

In this sample MVP proof to be a safe and effective embolic device, able to achieve definitive vessel occlusion without additional subsequent agents in 75% of the cases. The most frequently adopted models were in order MVP-5 and MVP-3 in bleeding patients: compared to other plugs, the main technological improvement of these devices is the possibility of releasing peripherally through a microcatheter. While eventual concomitant anticoagulation therapy did not influence the technical outcome, straight course and length of the landing zone are essential parameters to evaluate before deployment.

## Data Availability

The datasets supporting the conclusions of this article are available at Cardarelli Hospital of Naples Italy and Fondazione IRCCS Cà Granda Ospedale Maggiore Policlinico of Milan Italy RIS-PACS systems; for any questions, please contact the corresponding author.

## Abbreviations

AUC: Area under the curve
CIRSE: Cardiovascular and Interventional Radiology Society of Europe
CT: Computed tomography
DSA: Digital subtraction angiography
Fr: French
HCC: Hepatocellular carcinoma
MVP: Microvascular plug
PTFE: Polytetrafluoroethylene
RFA: Radiofrequency ablation
